# Production of biodiesel from non-edible feedstocks using environment friendly nano-magnetic Fe/SnO catalyst

**DOI:** 10.1038/s41598-022-20856-7

**Published:** 2022-10-06

**Authors:** Maryam Hanif, Ijaz Ahmad Bhatti, Muhammad Zahid, Muhammad Shahid

**Affiliations:** 1 Department of Chemistry, University of Agriculture, Faisalabad, 38040 Pakistan; 2 Department of Biochemistry, University of Agriculture, Faisalabad, 38040 Pakistan

**Keywords:** Catalysis, Chemical synthesis

## Abstract

Environmental problems associated with chemical catalysts to fulfil an ever-increasing energy demand have led to the search for an alternative environment friendly heterogeneous catalyst. If a catalyst being used in the biodiesel production is not environment friendly, then the environment is being contaminated in another way while trying to avoid pollution caused by burning of fossil fuels. The present study reports the use of nano-magnetic catalyst Fe/SnO supported on feldspar for the transesterification of various non-edible feedstocks oil, including *Pongamia* *pinnata* (karanja), *Carthamus* *oxyacantha* (wild safflower), *Citrullus* *colocynthis* (bitter apple), *Sinapis* *arvensis* (*wild* *mustard*) and *Ricinus* *communis* (castor). The optimized transesterification parameter was oil to methanol ratio (1:5, 1:10, 1:15, 1:20 and 1:25), catalyst amount (0.5, 1, 1.5, 2, 2.5%), temperature (40, 50, 60, 70 and 80 °C), and reaction times (30, 60, 90, 120 and 150 min). The biodiesel yield was found to be more than 97% for all the tested feedstocks with a maximum biodiesel yield of 98.1 ± 0.6% obtained for bitter apple seed oil under optimum conditions (oil to methanol ratio of 1:10, catalyst amount of 1% at 50 °C for 120 min). The catalysts used for transesterification were magnetically extracted after completion of the reaction. Different physico-chemical parameters like pour point, density, cloud point, iodine value, acid value, saponification and cetane number were determined and the quality of all the biodiesel samples were found to be in the standard range (ASTM D6751 and EN 1404). Different techniques like XRD, FTIR, SEM and EDX were used to characterize the prepared nano-magnetic (Fe/SnO/Feldspar) catalyst.

## Introduction

The rapid depletion of fossil fuel sources due to increase in global energy demand has promoted the need for alternative resources^1^. However, the alternative fuel should be renewable, economically feasible, easily available, and should have fewer environmental problems than conventional fuels^2^. Alternate renewable energy sources are helpful in overcoming the limitation of fossil fuels. Biodiesel is considered as a real alternative fuel for the conventional fuels because of its safe and renewable nature having minimal exhaust emission^3^. Bio-methanol can produce biodiesel via the transesterification process, where triglycerides/ lipids are transformed into fatty acid methyl ester using a catalyst and alcohol (methanol). The non-catalytic route for biodiesel production via subcritical and supercritical methanol is also reported in the literature^4^. Glycerol is produced as a byproduct with biodiesel during transesterification^5^. It is used in many industries such as botanical extracts, cosmetic industries, pharmaceutical, food industry, and chemical intermediates. During anaerobic digestion glycerol is used as a substrate^6^.

The main advantage and increasing concern for biodiesel production is availability of unlimited feedstocks. The most significant features of ideal feedstocks are high yield and low production cost. In the biodiesel industry selection of raw material is very important as 80% cost of biodiesel is associated with feedstock cost. Biofuel can be produced from edible feedstocks (like corn, rapeseed, palm oil and soybean) and non-edible feedstocks (like Karanja oil, Jatropha and Mahua)^7^. The edible plants as feedstocks compete with human food, freshwater and require large areas of fertile lands^8^. The production cost increases up to 70–92% due to increase in the cost of edible oils. Therefore, low-cost waste oils have been receiving more attention for biodiesel production as an appropriate candidate to replace diesel fuel in recent times.

In this work five non-edible oils such as *Pongamia* *pinnata* (Karanja), *Carthamus* *oxyacantha* (*Wild* *safflower*), *Sinapis* *arvensis* (*Wild* *mustard*), *Citrullus* *colocynthis* (bitter apple), and *Ricinus* *communis* (castor oil) were utilized for the biodiesel production. *Pongamia* *pinnata* is commonly known as karanja. Karanja is a hardy, perennial, medium sized glabrous tree that grows in many areas. Its seeds contain 30–40% extractable oil^9^. *Sinapis* *arvensis* (wild mustard) is a non-edible oil plant due to the presence of high erucic acid levels. It grows in calcareous soils in sunny places. The seeds contain approximately 34–45% extractable oil^10^. *Carthamus* *oxyacantha* (wild safflower) is a spiny leafed annual herb. It is a hardy and xerophytic poisonous weed crop. Its seed contain 25–32% oil (w/w)^11^. *Citrullus* *colocynthis* is generally known as bitter apple. It is a wild plant that grows in sandy soils that can bear the severe desert temperature. Its seeds contain up to 47% extractable oil and was discovered as potential source for biodiesel production^12^. The *Ricinus* *communis* (castor oil) grows in marginal soils. The mature seeds contain ricin (toxic protein) and are noxious to animals and humans. Castor oil yields between 40 and 60% oil. So it is explored as a new source for biodiesel production^13^. However, a major disadvantage of most non-edible oils is high content of FFAs, which raises the production cost of biodiesel.

The selection of catalyst is generally determined by nature of feedstock and the amount of FFA. Catalysts used for the biodiesel production are classified into two groups: homogeneous and heterogeneous. But the disadvantage of homogeneous catalysts is slow rate of reaction, high saponification value in feedstock that have high fatty acid contents, difficult product/catalyst separation and reusability issues. However, heterogeneous catalysts have many merits as compared to homogeneous catalysts such as low cost, easy separation, reusability, selectivity, and high rate of reaction. Because of these facts, heterogeneous catalyzed transesterification reactions are gaining significant importance^14^.

However, due to the high content of FFA, the processing of non-edible oils using base-catalytic processes is unfeasible. Pre-treatment of FFA content was very effective. Therefore, a two-step process is more effective in which esterification is done before the transesterification process. However, a major disadvantage of a two-step process is that it raises the production cost of biodiesel. To avoid the two-step process, the synthesis and use of bifunctional catalysts that have both acidic and basic active sites is more effective because it promotes the esterification as well as transesterification processes^15^.

The problems in the separation of catalysts from the reaction mixture significantly increases final product purification cost. These limitations can be overcome by the use of magnetic nanoparticles (MNPs) catalysts. The application of magnetic separation process avoids the loss of catalyst and catalyst reusability increases as compared to separation through centrifugation or filtration processes. Catalyst is quickly and easily separated when an external magnetic field is applied^16^. In magnetic nano-particles (MNPs) agglomeration arises due to magnetism which decreases the surface area of the catalyst^17,18^. Catalytic supports prevent sintering and agglomeration of small catalyst particles, revealing additional surface area. Clay materials, compared to other supports, have the advantages of abundance, low cost, environmental compatibility, high selectivity, reusability and operational simplicity^19^. Feldspar is the most abundant aluminosilicate clay mineral on the earth’s crust.

The main objective of the current study was the production of the biodiesel from non-edible oils (*Pongamia* *pinnata* (karanja), *Carthamus* *oxyacantha* (wild safflower), *Citrullus* *colocynthis* (bitter apple), *Sinapis* *arvensis* (*wild* *mustard*) and *Ricinus* *communis*(castor)) using novel magnetic iron/tin oxide (Fe/SnO) nanoparticles supported on feldspar. Specifically, the aim of this work was to: (1) synthesize the iron/tin (Fe/SnO) oxide nanoparticles supported on feldspar using wet impregnation method; (2) optimize various transesterification reaction parameters, (3) analytical characterization of biodiesel and catalyst.

## Materials and methods

### Chemical and reagent

Stannic chloride (SnCl_2_), Ferric Chloride (FeCl_3_), methanol (99%), ethanol (99.5%), Nitric acid, hydrochloric acid (37%), Sodium sulphate (anhydrous), sodium hydroxide (99%), feldspar, potassium hydroxide, petroleum ether, sodium thiosulphate, potassium iodide, phenolphthalein, starch and Wijs solution used during the present study were of analytical grade. Feldspar was collected from ceramic industry Gujranwala, Pakistan.

## Materials and oil extraction

*Pongamia* *pinnata* (karanja), *Carthamus* *oxyacantha* (wild safflower), *Citrullus* *colocynthis* (bitter apple), *Sinapis* *arvensis* (*wild* *mustard*) and *Ricinus* *communis* (castor oil) seeds were collected from wild areas of Pakistan. *Pongamia* *pinnata* (karanja), *Carthamus* *oxyacantha* (wild safflower), *Citrullus* *colocynthis* (bitter apple), *Sinapis* *arvensis* (*wild* *mustard*) and *Ricinus* *communis* (castor oil) were identified by Dr. Mansoor Hameed, Department of Botany, University of Agriculture, Faisalabad, Pakistan and was deposited to herbarium placed at Nano and Biomaterials Lab, Department of Chemistry, University of Agriculture, Faisalabad, Pakistan under the voucher specimen number 21-MH-001, 21-MH-002, 21-MH-003, 21-MH-004, and 21-MH-005, respectively.

The collected seeds were cleaned and stored in dark at room temperature till further processing. Oil was extracted from the ground seeds with the help of an automatic screw press machine (Vosoco oil press machine) in which ground seeds were squeezed under high pressure. The extracted oil was purified using vacuum filtration assembly to remove impurities and solid particles. The percentage oil contents of all the feedstock was calculated using Eq. (1)^20^.

1 $$ \% \;oil\;contents = \frac{Weight\;of\;oil\;obtained\;\left( g \right)}{{Weight\;of\;sample\;used\;\left( g \right)}} \times 100 $$

## Preparation of supported nano-magnetic catalyst

A solution of 2:1 ratio (v/v) of FeCl_3_ (0.5 mol L^−1^) and SnCl_2_ (0.5 mol L^−1^), respectively, was prepared. The obtained solution was immediately mixed with 1 L of NaOH (2 mol L^−1^) at 100 °C using constant stirring for 2 h. The appeared dark precipitates were first washed with distilled water and then treated with HNO_3_ (1 mol L^−1^). After a final washing of dark precipitates using the distilled water, these were filtered with whatman filter paper (No. 1). The nano particles were dried at 100 °C for 2 h and then activated thermally at a temperature of 300 °C for 4 h^21^. Prepared nanocatalysts were ground to a uniform particle size by using a pestle and mortar.

Supported nano-magnetic catalyst was prepared by mixing 0.5 g nano-magnetic catalyst (Fe/SnO) with 0.75 g of support (Feldspar) in deionized distilled water (DDW) to make a paste. The fine paste was then heated at 150 °C for an 1 h in the oven. Finally, a prepared support coated nano-magnetic catalyst was grounded and passed through a nano sieve to obtain uniform size nanoparticles.

## Characterization of catalyst

The structure of nano-magnetic catalysts was studied using the Shimadzu model XRD 6000 Power X-ray diffractometer using Cu-Kα radiation (40 kV and 30 mA) using wavelength (*λ*) of 1.5406 Å. Data were collected over a 2θ range from 15° to 70° with a step of 0.02° at speed 6 degree/minute. Crystallite size of material was calculated using Scherrer equation. FTIR spectra were obtained from 4000 to 650 cm^−1^ to study the functional group peaks (Agilent technologies, FTIR spectrometer). The structural properties of nanoparticles were characterized using SEM (NOVA NANOSEM-450), while powder samples spread on carbon tape were used to determine the elemental composition using EDX, Nova 450 at 5.00 kV.

## Transesterification

Transesterification was carried out in a 250 mL round bottom flask fitted to reflux condenser and heated on magnetic stirrer plates. The extracted vegetable oils of karanja, wild mustard, wild safflower, bitter apple, and castor seed oils were converted into fatty acid methyl ester (FAME) by catalytic transesterification using methanol in the presence of nano-magnetic catalyst (Fe/SnO/Feldspar). The extracted seed oils were weighted (50 g) and poured into a 250 mL round bottom flask. The mixture of oil, methanol and nano-magnetic catalyst were heated at 60 °C. After the reaction occurred completely, the catalyst was separated from the reaction system by using a permanent magnet. The separating funnel was used to separate the two layers. The upper layer contained methyl ester while glycerol was present in the bottom layer. The produced biodiesel was washed using excess hot (75–80 °C) distilled water to remove soap and surplus methanol. The % biodiesel yield was calculated by using Eq. (2)^22.^

2 $$ Process\;yield\;\left( \% \right) = \frac{Weight\;of\;biodiesel\;\left( g \right) }{{Weight\;of\;Oil\;\left( g \right)}} \times 100 $$

**Figure 1:**
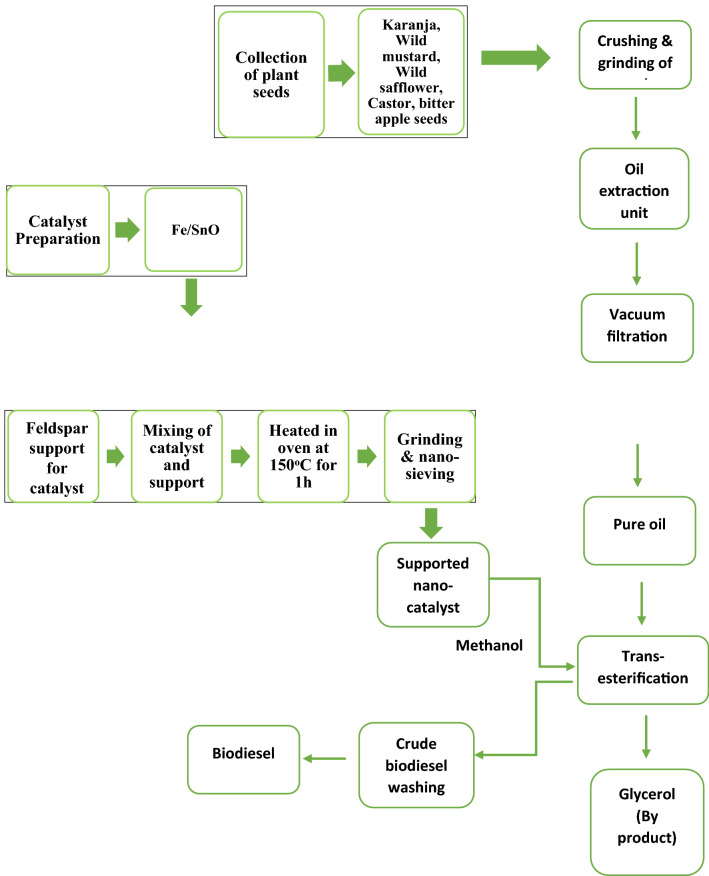
Schematic representation for biodiesel production from five different non-edible oils using Fe/SnO/Feldspar nano-magnetic catalyst.

The effect of various transesterification reaction parameters including methanol to oil molar ratio, catalyst concentration, reaction temperature, and reaction time were optimized during the present study to obtain maximum biodiesel yield as shown in Table 1. An extensive literature survey was made before the selection of the levels of the process parameters. A detail of the biodiesel production procedure is represented in Fig. 1.

**Table 1:** Transesterification reaction parameters.

Sr No	Reaction parameters	Reaction conditions
1	Catalyst concentration (wt%)	0.5%, 1%, 1.5%, 2%, 2.5%
2	Molar ratio (methanol/oil)	5:1, 10:1, 15:1, 20:1, 25:1
3	Temperature (°C)	40, 50, 60, 70, 80
4	Reaction time (min)	30, 60, 90, 120, 150

## Characterization of biodiesel

Gas-chromatographic mass spectrometric (GC–MS) analysis was performed to quantify FAME contents. Analyses were conducted using a Perkin-Elmer Clarus 500 model GCMS equipped with a capillary column (HP-1, 30 m × 0.25 mm × 0.25 µm), coupled to a Perkin-Elmer (Waltham, MA, USA) Clarus 500C MS. The sample injection tool was placed at an oven temperature of 50 °C and was held at that temperature for 1 min. The oven temperature was then increased to 325 °C at a heating rate of 10 °C/min and held for 2 min. Helium (99.99%) with a constant flow rate of 1.2 mL/min was used as a carrier gas. Unknown compounds were identified by comparing GC–MS peaks of compounds with mass spectrums libraries of NIST.

## Quality parameters of biodiesel

Different physicochemical parameters like density, pour point (PP), cloud point (CP), iodine value (IV), saponification value (SV), acid value (AV), and cetane number (CN) of biodiesel were measured according to the method described in the previous literature^23^.

## Results and discussion

### Seed oil yield (%)

The percentage oil yield of 20 kg seeds of castor, karanja, wild mustard, wild safflower, and bitter apple was 39.2%, 37.05%, 32.5%, 29.55%, and 17.95%, respectively.

### XRD analysis

**Figure 2:**
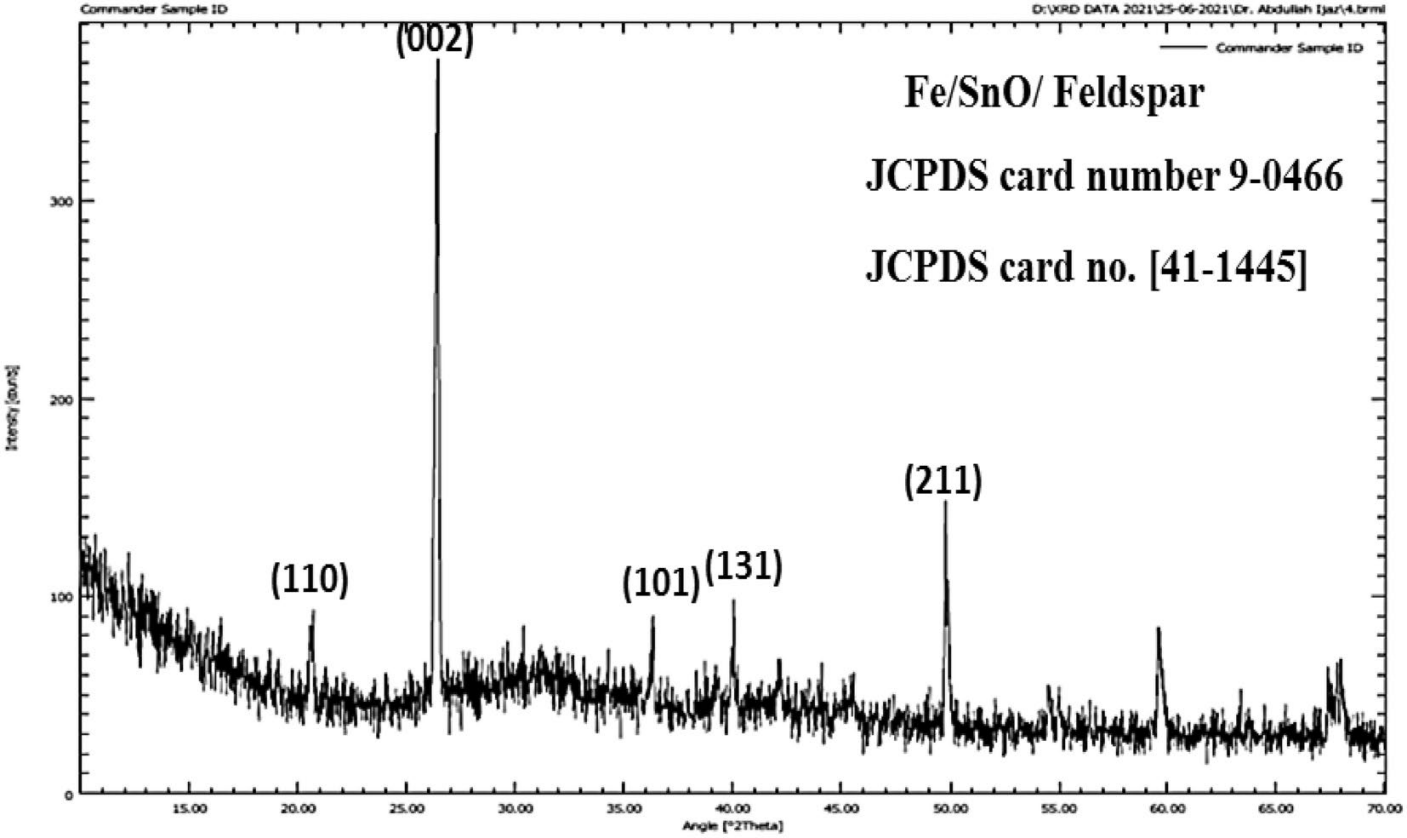
XRD of Fe/SnO/Feldspar.

The XRD pattern of Fe/SnO_2_/Feldspar is shown in Fig. 2. Diffraction peaks show tetragonal structure of SnO_2_ nanoparticles in the JCPDS card no. [41-1445]. The high intensity peaks at 20.6°, 36.3° and 50° are associated with (110), (101) and (211) reflection planes of iron tin oxide. The peaks at 2θ = 26.41°, 40°, 59.60° and 67.76° appeared due to the presence of feldspar. According to Debye–Scherrer equation the average crystal size of Fe/SnO_2_/Feldspar nano-magnetic catalyst is 31.178 nm (Table 2). The XRD of pure feldspar has been previously study by our research group^24^. The silica and alumina comprised most of the pure feldspar composition. The particle size of feldspar clay measured using X-Rays was 47.58 nm^24^.

**Table 2:** XRD spectral analysis details of Fe/SnO/Feldspar (supported nano-magnetic) catalyst.

Position (2θ)	FWHM Left (2θ)	Area (cts. 2θ)	Background (cts)	d-Spacing (Å)	Height (cts)	Relative intensity (%)	Particle size (nm)	Average particle size
20.624	0.236	6.66	46.59	4.30661	28.6	8.57	32.619	31.178
26.413	0.098	32.41	48.88	3.37439	333.86	100	61.138	
36.315	0.118	4.87	45.3	2.47383	41.79	12.52	37.050	
40.006	0.236	6.46	42.64	2.25371	27.73	8.31	16.816	
49.786	0.072	9.91	33.03	1.83	103.2	30.91	44.329	
59.605	0.118	6.12	28.33	1.55112	52.55	15.74	22.573	
67.768	0.629	12.54	27.71	1.3828	20.19	6.05	3.72308	

### FTIR analysis

**Figure 3:**
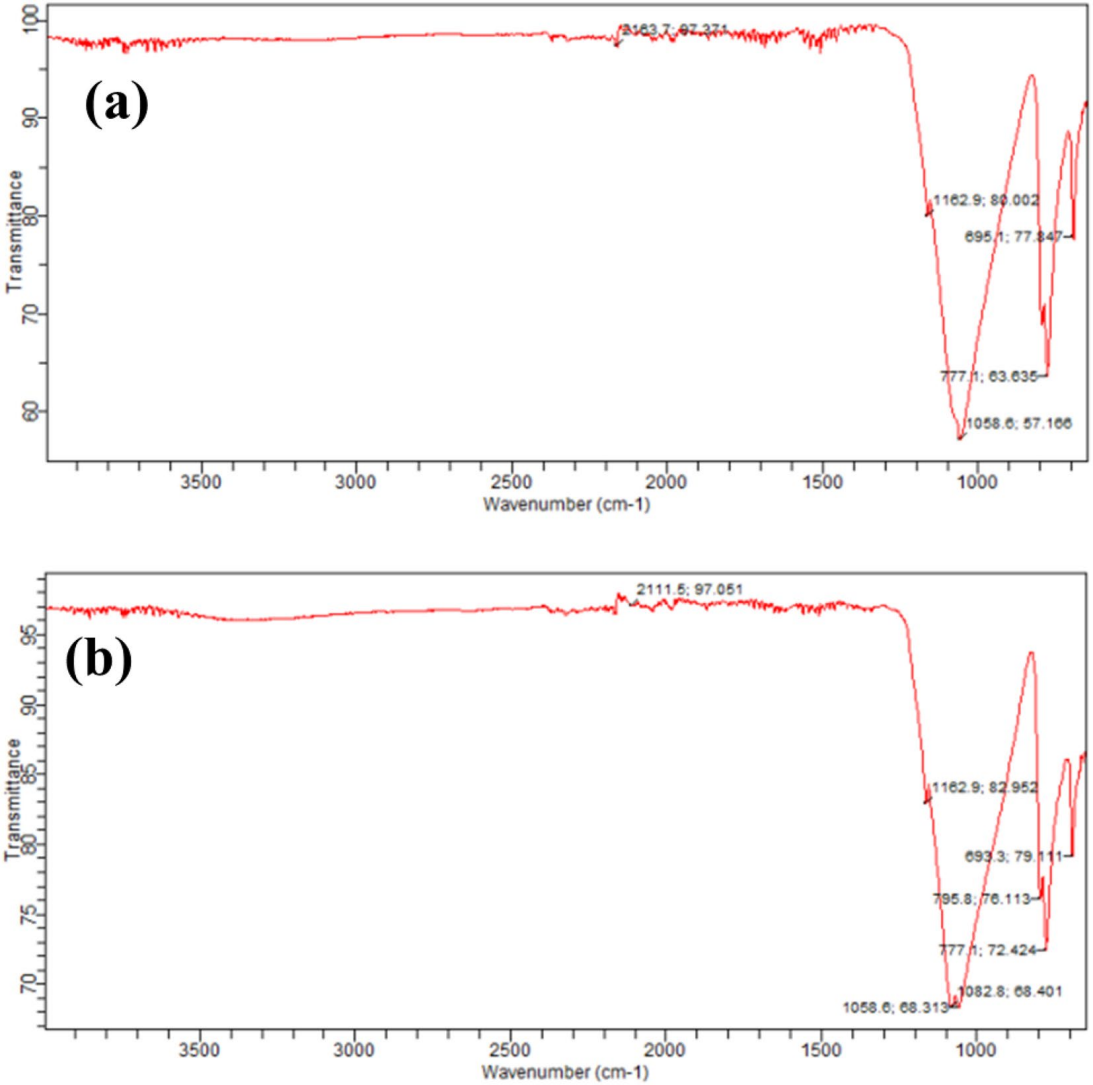
FTIR spectra of (**a**) Feldspar (catalyst support) (**b**) Fe/SnO/Feldspar.

Feldspar is an aluminosilicate clay mineral. The untreated feldspar absorption peaks at 695 cm^–1^ appeared due to the silicon-oxygen bending vibrations. The peak around 777 cm^−1^ is correlated to Si–O–Si stretching vibration of quartz. The strong and broad band at 1058 cm^−1^ was due to the Si–O–Al stretching vibrations which proved the aluminosilicate type of this sample (Fig. 3a). The small peak at 2163.7 cm^−1^ was due to H–O–H bending vibration. Similarly in a previous study the Si–O–Al peak was located at ~ 1058 cm^−1^. Previously, SiO_2_ bearing bonds such as Si–O–Si were located approximately at around ~ 776 cm^−1^ ^25^. The FTIR of Fe/SnO/Feldspar is shown in Fig. 3b. Previously very strong absorption peaks observed in the range of 420–700 cm^−1^ were attributed to the Sn–O antisymmetric vibrations^26^. Therefore, the peak at 693 cm^−1^ was due to Sn–O vibration. The absorption peak at 777.1 cm^−1^ was correlated to Si–O–Si stretching vibration of quartz. The strong peaks at 1082 cm^−1^ and 1058 cm^−1^ appeared due to the Si–O–Al stretching vibrations which showed the successful loading of the catalyst on feldspar support. The absorption peak at 2111.5 cm^−1^ was assigned to the vibration of hydroxyl group due to the absorbed/adsorbed water and showed a stretching vibrational mode of O–H group.

### SEM/EDX analysis

**Figure 4:**
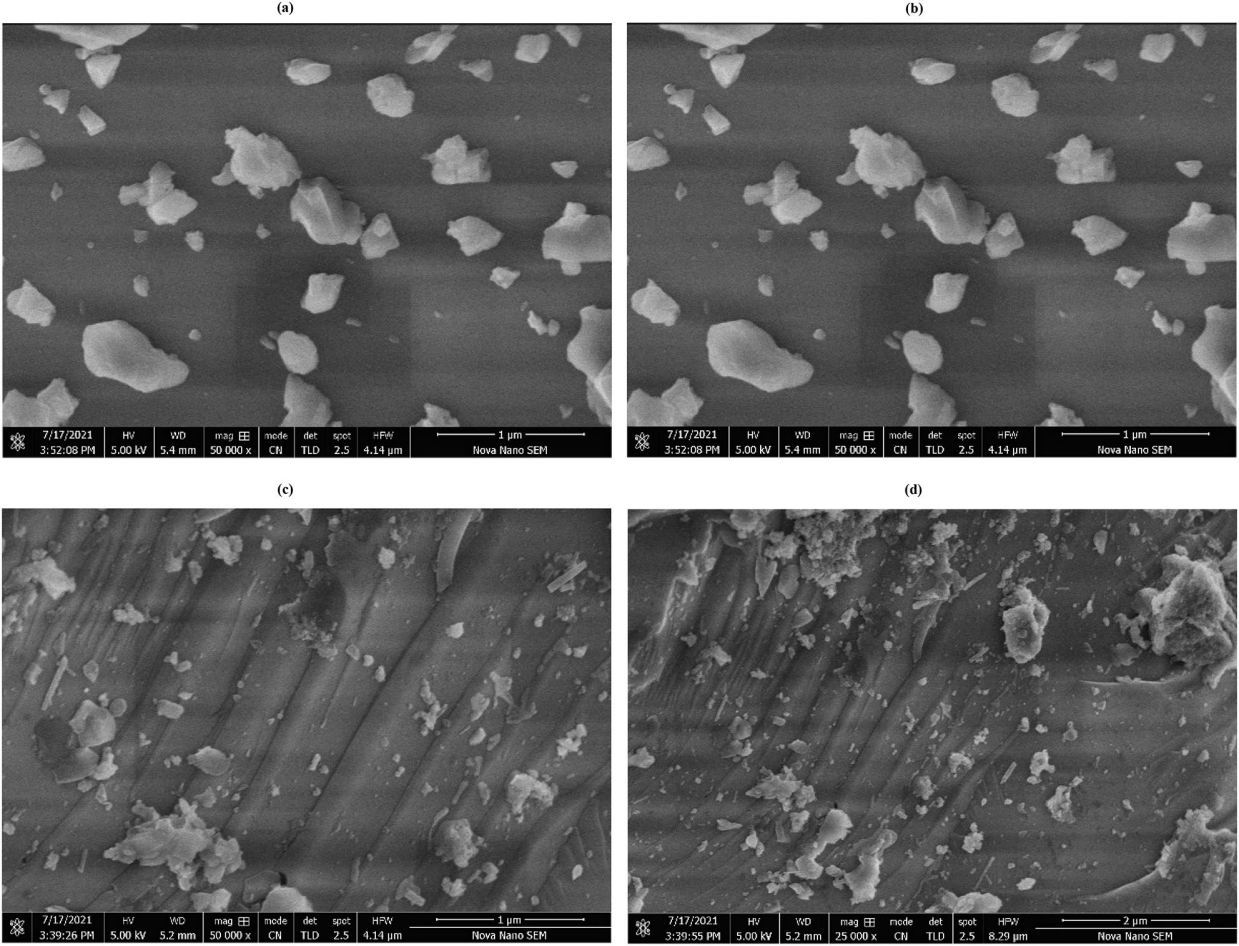
SEM images of (**1a**) Feldspar at 1 µm (**1b**) Feldspar at 2 µm; (**2a**) Fe/SnO/Feldspar at 1 µm (**2b**) Fe/SnO/Feldspar at 2 µm.

The SEM images of feldspar are shown in Fig. 4a–d. The micrograph of feldspar has shown that sample particles were of high crystallinity with non-uniform size. These SEM images also showed that feldspar had a lamellar-like mesoporous structure with a regular inclination. The lamellar structure gives high surface area to support for catalyst anchoring.

**Figure 5:**
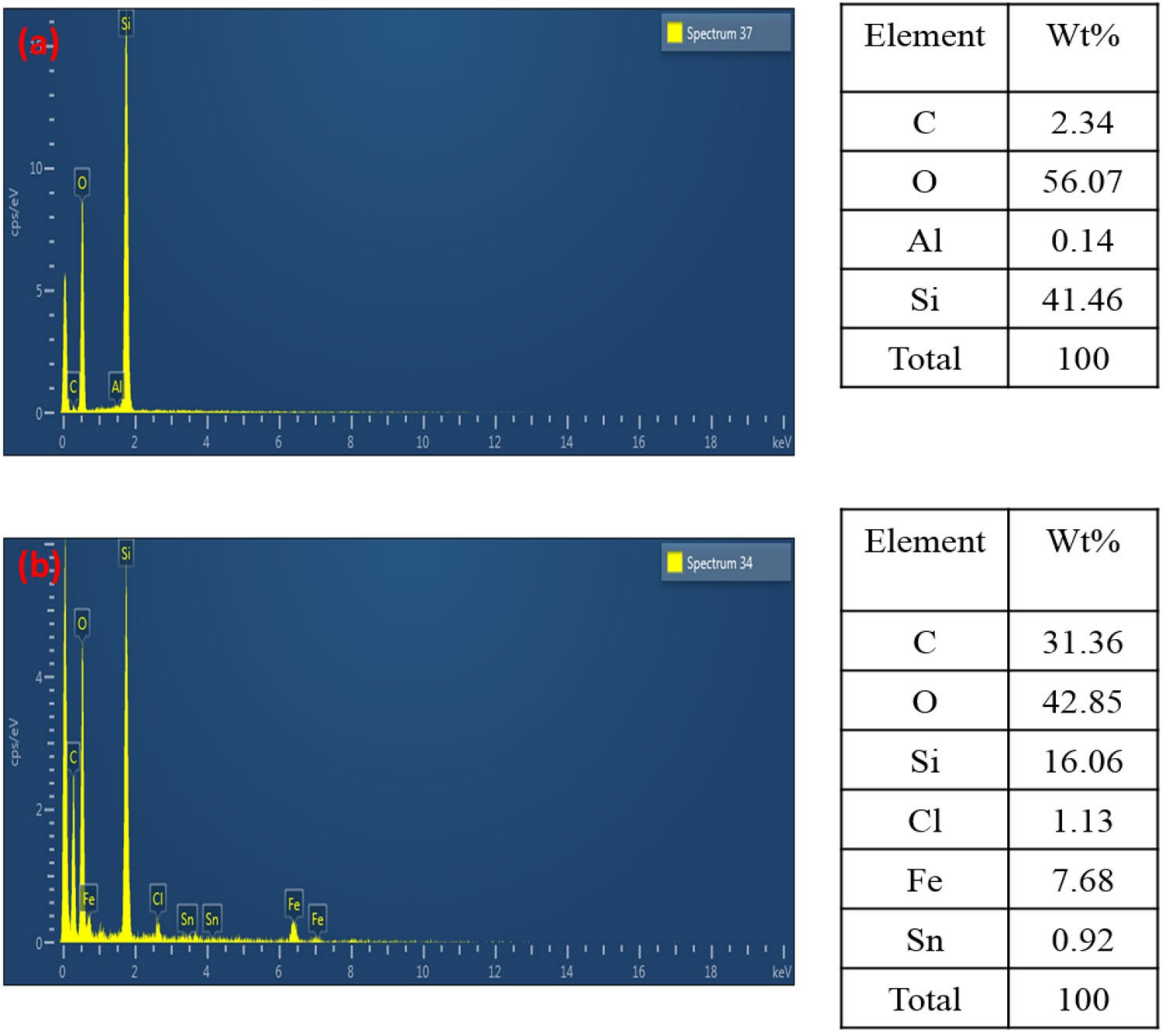
EDX of (**a**) Feldspar (catalyst support) (**b**) Fe/SnO/Feldspar.

Figure 5a demonstrates the results of EDX analysis of feldspar. Major components of feldspar are oxygen (O), silicon (Si) and aluminium (Al) with weight percentage of 56.7%, 41.46% and 0.14% respectively. These results confirmed the aluminosilicate type of this sample^27^.

Figure 4c,d shows the microstructure and morphology of the fabricated Fe/SnO/Feldspar nano-catalyst visualized by using SEM. The micrograph shows wrinkle and crumples like-sheets morphology due to the presence of feldspar. The Fe/SnO particles are embedded on the wrinkly surface. The Fe/SnO catalyst exhibited spherical and non-spherical shape particles of uneven size due to agglomeration of iron particles in the sample^28^. This confirms the support of Fe/SnO on feldspar.

The EDX spectrum indicates the presence of oxygen (O), silicon (Si), iron (Fe) and tin (Sn) with the weight percentage of 42.85%, 16.06%, 7.68%, and 0.92%, respectively (Fig. 5b) confirming a good synthesis of the supported nano-magnetic catalyst (Fe/SnO supported on feldspar). The traces of carbon were also observed and can be attributed to the sample stub.

### Transesterification

Fe/SnO/Feldspar has been screened out as a most effective transesterification catalyst for various low grade (FFA) feedstocks oils and recorded biodiesel yields were more than 97% for all the tested feedstocks used in the present study. The maximum reaction yield for transesterification using non-edible seeds oils; karanja, wild mustard, wild safflower, bitter apple, and castor plant seed oils is shown in (Table 8). A previous study reported the biodiesel production from date seed oil using Fe_3_O_4_ nanoparticles loaded with the mercaptoacetic acid magnetic catalyst that gave 91.4% biodiesel yield^29^. In another study, biodiesel yield from waste loquat seed oil using bi-functional catalytic system (CaO/CeO_2_) was 90.14%^30^. The yield of biodiesel reported from another previous study using castor oil as non-edible feedstock in the presence of NaY zeolite-supported La_2_O_3_ catalyst was observed to be 84.6%^31^. Fe/SnO/Feldspar catalyst used in the present study produced biodiesel in much better yields. Fe/SnO/Feldspar catalyst was found to be an active catalyst for transesterification of non-edible oils (containing large numbers of free fatty acids) as this catalyst has weak and medium acidity regions (that can be Lewis or Brönsted acid sites)^21^. On the completion of the reaction, the catalyst was magnetically extracted for further use. In a previous study, it has been reported that Fe/SnO catalyst was used 4 times for the biodiesel production^21^. Fe/SnO/Feldspar catalyst used in the present study was 5 times successively employed for the biodiesel production without any loss in the yield.

### Optimization of transesterification process parameters

The effect of various parameters on the yield of biodiesel from the non-edible oils (karanja, wild mustard, wild safflower, bitter apple, and castor oils) in the presence of Fe/SnO/Feldspar nano catalyst is summarized in the Tables 3, 4, 5, 6 and 7. The effect of catalyst concentration was investigated at five concentration levels varying from 0.5 to 2.5% while keeping other reaction parameters constant at: reaction temperature of 40 °C, reaction time of 120 min and molar ratio of methanol to oil at 1:5. It was observed that karanja and castor oil with 1.5wt.% concentration resulted in highest biodiesel yields of 89.6 ± 0.1% and 90.0 ± 0.3%, respectively. However, the maximum biodiesel yield for wild mustard, wild safflower, and bitter apple was 87.8 ± 0.7%, 89.2 ± 0.7% and 86.8 ± 0.4%, respectively observed at 1wt% catalyst. The catalyst concentration optimized depends on the type and nature of the catalyst as well as on the fatty acid profile of oil used to produce biodiesel. As the catalyst concentration increased, the conversion of triglyceride, as well as the ester contents also increased^32^. Insufficient amount of catalyst resulted in incomplete conversion of triglycerides into the esters as indicated from its lower ester contents^33^. Lower biodiesel yields were observed at high concentrations of catalysts as this resulted in the production of highly concentrated mixture which restricted free movement of molecules^34,35^.

**Table 3:** Transesterification reaction parameters for karanja seed oil.

Feedstock	Conc. of catalyst (%)	Methanol to oil ratio	Temperature (°C)	Reaction time (min)	Biodiesel yield (%)
Karanja	0.5	5:1	40	120	85.6 ± 0.2
	1.00	5:1	40	120	86.0 ± 0.7
	1.5	5:1	40	120	89.6 ± 0.1
	2.00	5:1	40	120	88.5 ± 0.9
	2.5	5:1	40	120	87.0 ± 0.8
	1.5	10:1	40	120	90.5 ± 0.8
	1.5	15:1	40	120	86.6 ± 0.6
	1.5	20:1	40	120	84.1 ± 0.7
	1.5	25:1	40	120	83.0 ± 0.9
	1.5	10:1	50	120	97.4 ± 0.6
	1.5	10:1	60	120	93.0 ± 0.5
	1.5	10:1	70	120	92.5 ± 0.9
	1.5	10:1	80	120	89.0 ± 0.8
	1.5	10:1	50	30	90.1 ± 0.8
	1.5	10:1	50	60	93.5 ± 0.3
	1.5	10:1	50	90	94.3 ± 0.7
	1.5	10:1	50	150	95.1 ± 0.6

**Table 4:** Transesterification reaction parameters for wild mustard seed oil.

Feedstock	Conc. of catalyst (%)	Methanol to oil ratio	Temperature (°C)	Reaction time (min)	Biodiesel yield (%)
Wild mustard	0.5	5:1	40	120	85.0 ± 0.4
	1.00	5:1	40	120	87.8 ± 0.7
	1.5	5:1	40	120	86.1 ± 0.9
	2.00	5:1	40	120	84.0 ± 0.7
	2.5	5:1	40	120	83.3 ± 0.8
	1.00	10:1	40	120	85.1 ± 0.6
	1.00	15:1	40	120	83.0 ± 0.5
	1.00	20:1	40	120	82.2 ± 0.7
	1.00	25:1	40	120	80.5 ± 0.6
	1.00	5:1	50	120	94.0 ± 0.8
	1.00	5:1	60	120	92.0 ± 0.6
	1.00	5:1	70	120	89.0 ± 0.5
	1.00	5:1	80	120	86.0 ± 0.6
	1.00	5:1	50	30	90.0 ± 0.5
	1.00	5:1	50	60	93.6 ± 0.1
	1.00	5:1	50	90	97.6 ± 0.8
	1.00	5:1	50	150	91.4 ± 0.6

**Table 5:** Transesterification reaction parameters for wild safflower seed oil.

Feedstock	Conc. of catalyst (%)	Methanol to oil ratio	Temperature (°C)	Reaction time (min)	Biodiesel yield (%)
Wild safflower	0.5	5:1	40	120	88.0 ± 0.6
	1.00	5:1	40	120	89.2 ± 0.7
	1.5	5:1	40	120	87.0 ± 0.4
	2.00	5:1	40	120	86.0 ± 0.6
	2.5	5:1	40	120	85.2 ± 0.8
	1.00	10:1	40	120	87.1 ± 0.4
	1.00	15:1	40	120	84.0 ± 0.8
	1.00	20:1	40	120	82.4 ± 0.7
	1.00	25:1	40	120	79.9 ± 0.2
	1.00	5:1	50	120	98.1 ± 0.6
	1.00	5:1	60	120	89.5 ± 0.5
	1.00	5:1	70	120	85.0 ± 0.5
	1.00	5:1	80	120	83.4 ± 0.5
	1.00	5:1	50	30	89.7 ± 0.5
	1.00	5:1	50	60	93.4 ± 0.8
	1.00	5:1	50	90	95.1 ± 0.5
	1.00	5:1	50	150	96.6 ± 0.7

**Table 6:** Transesterification reaction parameters for bitter apple seed oil.

Feedstock	Conc. of catalyst (%)	Methanol to oil ratio	Temperature (°C)	Reaction time (min)	Biodiesel yield (%)
Bitter apple	0.5	5:1	40	120	85.1 ± 0.9
	1.00	5:1	40	120	86.8 ± 0.4
	1.5	5:1	40	120	84.5 ± 0.8
	2.00	5:1	40	120	83.2 ± 0.6
	2.5	5:1	40	120	82.0 ± 0.7
	1.00	10:1	40	120	88.0 ± 0.8
	1.00	15:1	40	120	85.2 ± 0.7
	1.00	20:1	40	120	84.4 ± 0.5
	1.00	25:1	40	120	82.1 ± 0.9
	1.00	10:1	50	120	98.5 ± 0.5
	1.00	10:1	60	120	90.1 ± 0.9
	1.00	10:1	70	120	87.0 ± 0.3
	1.00	10:1	80	120	85.4 ± 0.7
	1.00	10:1	50	30	87.7 ± 0.8
	1.00	10:1	50	60	91.6 ± 0.5
	1.00	10:1	50	90	94.7 ± 0.8
	1.00	10:1	50	150	95.6 ± 0.6

**Table 7:** Transesterification reaction parameters for castor seed oil.

Feedstock	Conc. of catalyst (%)	Methanol to oil ratio	Temperature (°C)	Reaction time (min)	Biodiesel yield (%)
Castor	0.5	5:1	40	120	85.2 ± 0.9
	1.00	5:1	40	120	88.0 ± 0.8
	1.5	5:1	40	120	90.0 ± 0.3
	2.00	5:1	40	120	87.0 ± 0.2
	2.5	5:1	40	120	86.4 ± 0.9
	1.5	10:1	40	120	91.1 ± 0.8
	1.5	15:1	40	120	92.0 ± 0.3
	1.5	20:1	40	120	89.2 ± 0.5
	1.5	25:1	40	120	87.0 ± 0.6
	1.5	15:1	50	120	92.1 ± 0.5
	1.5	15:1	60	120	97.0 ± 0.6
	1.5	15:1	70	120	92.1 ± 0.7
	1.5	15:1	80	120	89.5 ± 0.2
	1.5	15:1	60	30	88.6 ± 0.5
	1.5	15:1	60	60	92.1 ± 0.7
	1.5	15:1	60	90	93.6 ± 0.8
	1.5	15:1	60	150	94.6 ± 0.4

The effect of methanol to oil ratio was investigated for five different levels (5:1, 10:1, 15:1, 20:1 and 25:1 methanol: oil) to optimize biodiesel yield by keeping the catalyst amount fixed at 1.5wt.% for karanja and castor oil while for wild mustard, wild safflower, and bitter apple at 1wt%. For karanja and bitter apple oils, 10:1 methanol to oil ratio was adequate to complete the conversion of triglycerides into methyl esters. It can be seen from results that on increasing methanol to oil ratio from 10:1 to 15:1, the yield of the biodiesel product dropped from 90.5 ± 0.8% to 86.6 ± 0.6% and 88.0 ± 0.8% to 85.2 ± 0.7% for karanja and bitter apple oils, respectively. For wild mustard and wild safflower, the optimal methanol to oil was 5:1, which produced a maximum ester content of 87.8 ± 0.7%, and 89.2 ± 0.7%, respectively. While the castor oil showed the highest biodiesel yield (92.0 ± 0.3%) at 15:1. The various feedstock oils showed different optimized methanol to oil ratio due to difference in viscosity of oils and the viscosity of methanol to oil mixtures changed after adding the catalyst. Castor oil has higher viscosity as compared to other vegetable oils^36^. To produce biodiesel from the castor oil, it has been found that a higher methanol/oil ratio was required to improve the contact between methanol and oil molecules. Therefore, the optimum methanol/oil ratio was 5:1 (for wild mustard and wild safflower), 10:1 (for karanja and bitter apple) and 15:1 (for castor oil). At the lower molar ratio in comparison to the optimum level, the biodiesel yield was found to be low because of the incomplete conversion of reactants into product. However, at higher molar ratio, the obtained biodiesel yield was low as methanol forms emulsion^37^.

The impact of temperature on methyl ester yield (Tables 3, 4, 5, 6 and 7) was investigated on five temperatures (40, 50, 60, 70 and 80 °C) by keeping all other reaction parameters constant as mentioned in the previous section. The maximum biodiesel yield for karanja, wild mustard, wild safflower, and bitter apple oils were 97.4 ± 0.6%, 94.6 ± 0.8%, 98.1 ± 0.6% and 98.5 ± 0.5%, respectively at 50 °C. While castor oil exhibited maximum biodiesel of 97.0 ± 0.6% at 60 °C. Castor oil had higher viscosity than other studied oils. The rise in reaction temperature could lead to decrease in viscosity and increase in effective collision among the reacting molecules. However, temperature above the optimum level decreased the ester yield may be due to the evaporation of methanol from reaction mixture^38^.

To determine the optimum reaction time for the maximum biodiesel yield, the reactions were conducted at varying reaction time (30 to 150 min) by keeping other reaction parameters constant. It was found that karanja, wild safflower, bitter apple and castor oils gave the highest biodiesel yield after 120 min of reaction. The maximum biodiesel yield for karanja, wild safflower bitter apple and castor oil was 97.4 ± 0.6%, 98.1 ± 0.6%, 98.5 ± 0.5% and 97.0 ± 0.6%, respectively. However, wild mustard oil produced the highest biodiesel yield i.e., 97.6 ± 0.8% after 90 min of reaction time. The maximum yield of biodiesel could be associated at different reaction times for various feedstocks to the molecular structure of the oil’s saturated fatty acids. The oils having higher contents of saturated fatty acids require a longer period for heating as they have higher activation energy. Overall optimum transesterification reaction conditions for the five different oil samples (Karanja, Wild mustard, Wild safflower, Castor, and Bitter apple oils) and their maximum ester yield using Fe/SnO/Feldspar catalyst is shown in Table 8.

**Table 8:** Optimal transesterification reaction conditions for different non-edible oils using Fe/SnO/Feldspar catalyst.

Feedstock	Optimal reaction conditions				Yield (%)
	Catalyst loading (wt% to the oil)	Methanol to oil molar ratio	Temperature (°C)	Time (min)	
Karanja	1.5	10:1	50	120	97.4 ± 0.6
Wild mustard	1	5:1	50	90	97.6 ± 0.8
Wild safflower	1	5:1	50	120	98.1 ± 0.6
Bitter apple	1	10:1	50	120	98.5 ± 0.5
Castor	1.5	15:1	60	120	97.0 ± 0.6

### Assessment of fuel quality parameters

Biodiesel properties of karanja, wild mustard, wild safflower, bitter apple, and castor oils using Fe/SnO/Feldspar catalyst were determined and compared against diesel, and the American biodiesel standard, ASTM D6751 (Table 9). Density is an important property of fuel as fuel injection systems, fuel pumps, and fuel injectors must provide a precise amount of fuel for a proper combustion. Biodiesel with high density could produce incomplete combustion while low density fuel could be highly volatile. Biodiesel density depends on the nature of feedstock, method used to produce biodiesel and fatty acids methyl ester profile^39^. The American standard (ASTM D6751), does not define any density specification limits^40^. The recommended range of density lies between 0.86 and 0.90 g/ml by EN 14214:2003 for a B100 type biodiesel. In the present research, the density of all the biodiesel samples were found to be in the standard range of 0.87–0.88 g/ml for karanja, wild mustard, wild safflower, bitter apple, and castor oils. Cloud point (CP) is the temperature at which the wax crystals first appear to give it a cloudy appearance. Biodiesel CP is related to the fatty acid composition of feedstock. CP decreases with higher degree of unsaturation and increases with fatty acid chain length increase. Pour point (PP) is the minimum temperature of any fuel at which it loses its flow characteristics. PP is also an important parameter in determining the cold flow operation since the biodiesel is suitable for operation only above the pour point value. Generally, The CP and PP of biodiesel are higher as compared to conventional diesel^41^. CP (− 3 to 15 °C) and PP (− 5 to 10 °C) values obtained during the present study were in the range described by the ASTM^42^ (Table 9).

**Table 9:** Comparison of fuel properties of FAME from karanja, wild mustard, wild safflower, bitter apple, and castor seed oils with diesel.

Fuel parameters	Karanja	Wild mustard	Wild safflower	Bitter apple	Castor oil	Diesel ASTM D975	ASTM D6751 Limits
Density (g/ml)	0.87	0.88	0.87	0.88	0.87	0.85	Not specified
Cloud point (˚C)	3.1	− 3	1.5	0.1	0.3	− 15 − 5	− 3 to 15
Pour point (˚C)	− 1.3	− 1.1	− 3.4	− 4.4	− 2.2	− 35 to 15	− 5 to 10
Acid value (mg KOH/g)	0.14	0.41	0.42	0.14	0.15	–	0.50 max
Iodine value (g I_2_/100 g)	80.3	81.24	65.97	97.87	87.72	–	Not specified
Saponification value (mg KOH g^–1^ oil) oil)	175.01	179.04	194.06	197.03	180.02	–	Not specified
Cetene number	59.41	58.50	59.58	51.98	56.88	40–55	47 minimum

Acid value is a measure of the free fatty acids (FFA) present in the fat/oil. According to ASTM standard D6751, the maximum allowed level of AV in pure biodiesel is 0.8 mg KOH g^–1^ ^43^. Biodiesel AV (mg KOH g^–1^) for karanja, wild mustard, wild safflower, bitter apple, and castor oils were 0.14, 0.41, 0.42, 0.14 and 0.15, respectively. Iodine value (IV) determines the degree of unsaturation present in the oil. Low iodine value biodiesel is more efficient fuel and is easily combustible than those with higher iodine values that may exhibit poor cold flow properties. The oxidation stability of oil is dependent upon the degree of unsaturation. On heating, biodiesel having higher amounts of unsaturated fatty acids undergoes polymerization and results in formation of deposit and fuel lubrication deterioration. According to the EN14214 biodiesel standard, a maximum of 120 g I_2_/100 g of biodiesel iodine value is acceptable^44^. ASTM specified no limits for iodine value. Iodine value decreases with chain length contrary to increase with degree of unsaturation^3^. Unsaturation in biodiesel is required to some degree to avoid its solidification. Biodiesel cloud point (CP) and pour point (PP) are also dependent on iodine value. Higher the degree of unsaturation (high iodine value), the lower will be CP. Iodine value of biodiesel produced using karanja, wild mustard, wild safflower, bitter apple, and castor oils biodiesel were 80.3, 81.24, 65.97, 97.87 and 87.72 g I_2_/100 g, respectively.

Saponification is a process that involves production of soap or metal salts from lipids. Saponification value (SV) is the indication of the amount of saponifiable units (acyl groups) per unit weight of oil. A low SV indicates a higher proportion of high molecular weight fatty acids in the fat/oil or vice versa. The SV is also used for determining the average molecular weight of fat/oil. The SV is expressed in milligrams of potassium hydroxide (mg KOH g^–1^ oil). Saponification value (mg KOH g^–1^ oil) of biodiesel produced using karanja, wild mustard, wild safflower, bitter apple, and castor oils were 175.01, 179.04, 194.06, 197.03 and 180.02, respectively. Cetane number (CN) is a crucial parameter that directly affects the ignition delay period. Fuels with higher CN can auto ignite in a short time after injection to the combustion chamber. Lower CN produces increased exhaust emission, higher knocking, abundant deposits in the engine because of incomplete combustion. Biodiesel CN increases with degree of saturation, chain length of fatty acids and higher oxygen contents. The CN of biodiesel fuel is specified by ASTM D613 (47 minimum) and EN ISO 5165 (51 minimum)^41^. In the present study, the maximum cetane number was 58.97 of the biodiesel sample synthesized by the wild safflower oil while the minimum cetane number was 53.21 of the biodiesel sample produced by the bitter apple oil. From the obtained results, it was observed that the cetane number measured for the produced FAME is higher than the minimum limits set by ASTM and EN standards (Table 9).

### Fatty acid profile

Biodiesel is a mixture of long chain FA with the number of C-atoms present in the chain varying from 14 to 22^45^. The fatty acid compositions of various oils used in the present study is given in Table 10. The major fatty acid present in karanja^46^, wild mustard^23^, wild safflower^47^, castor^36^ and bitter apple^48^ oils were oleic acid (51.92%), erucic acid (41.43%), linoleic acid (75.17%), ricinoleic acid (80.54%) and linoleic acid (70.71%), respectively. The results obtained clearly show that the feedstock oils used in the present study have significantly different fatty acid compositions.

**Table 10:** Fatty acid composition of various oils used in the present study.

Sr. No	Fatty acid	Molecular formula	Fatty acid amount (%)				
			Karanja oil	Wild mustard oil	Wild safflower oil	Castor oil	Bitter apple oil
1	Capric acid	C_10_H_20_O_2_	0.11	0.15	0.13	0.12	0.07
2	Lauric acid	C_12_H_24_O_2_	0.22	0.12	0.09	0.08	0.06
3	Myristic acid	C_14_H_28_O_2_	0.93	0.18	0.16	0.11	0.13
4	Palmitic acid	C_16_H_32_O_2_	10.33	3.63	7.73	1.30	8.35
5	Margaric acid	C_17_H_34_O_2_	0.09	0.05	0.06	0.07	0.01
6	Linolenic acid	C_18_H_30_O_2_	3.15	0.09	0.32	1.57	0.17
7	Linoleic acid	C_18_H_32_O_2_	11.03	15.75	75.17	7.65	70.71
8	Oleic acid	C_18_H_34_O_2_	51.92	23.11	12.98	5.83	9.96
9	Ricinoleic acid	C_18_H_34_O_3_	–	–	–	80.54	0
10	Stearic acid	C_18_H_36_O_2_	4.66	1.15	0.89	1.43	8.29
11	Eicosanoic acid	C_20_H_40_O_2_	9.76	12.83	0.11	0.18	0.03
12	Arachidic acid	C_20_H_40_O_2_	0.96	0.07	0.76	0.21	0.11
13	Erucic acid	C_22_H_42_O_2_	–	41.43	–	–	0.17
14	Behenic acid	C_22_H_44_O_2_	4.36	0.09	0.43	0.17	0.07
15	Lignoceric acid	C_24_H_48_O_2_	2.12	1.12	0.32	0.15	1.13

## Conclusions

The novel nano-magnetic iron doped tin oxide catalyst supported on feldspar (Fe/SnO/Feldspar) was prepared and tested as an effective catalyst for biodiesel synthesis using non-edible oils feedstock having quite variable fatty acid profile. The Fe/SnO/Feldspar nano-magnetic catalyst was found as the most effective transesterification catalyst for various feedstock oils used in present study. The biodiesel yield was found to be more than 97% for all the tested feedstocks with a maximum biodiesel yield of 98.1 ± 0.6% obtained for bitter apple seed oil under optimum conditions (oil to methanol ratio of 1:10, catalyst amount of 1% at 50 °C for 120 min). XRD, FTIR, SEM and EDX spectrum showed the successful loading of catalyst (Fe/SnO) on feldspar support. The XRD spectrum showed the average crystal sizes 41.83 nm and 31.17 nm for feldspar (support) and Fe/SnO nano-magnetic catalyst, respectively. All the biodiesel samples were found to be in the standard range specified in ASTM D6751 and EN 1404 standards. The results of the present study clearly demonstrated that the Fe/SnO/Feldspar nano-magnetic catalysts could be used as low cost and environmentally safe materials to produce biodiesel as compared to traditionally employed catalysts. Further pilot scale studies could be done for highlighting commercial importance of catalyst.

*Experimental* *research* *and* *permission* *statement*: It is submitted that the experimental research on plants, including the collection of plant material, complied with relevant institutional, national, and international guidelines and legislation. The plant collection procedures/permission and all other protocols were approved by Scrutiny committee of Faculty of Sciences, University of Agriculture, Faisalabad, Pakistan.

## Data Availability

All data has been included in the manuscript. If any other data required relevant to publication, corresponding author (Maryam Hanif) would provide that.
